# Protective Effect of *Artemisia asiatica* Extract and Its Active Compound Eupatilin against Cisplatin-Induced Renal Damage

**DOI:** 10.1155/2015/483980

**Published:** 2015-10-11

**Authors:** Jun Yeon Park, Dahae Lee, Hyuk-Jai Jang, Dae Sik Jang, Hak Cheol Kwon, Ki Hyun Kim, Su-Nam Kim, Gwi Seo Hwang, Ki Sung Kang, Dae-Woon Eom

**Affiliations:** ^1^College of Korean Medicine, Gachon University, Seongnam 461-701, Republic of Korea; ^2^Department of Surgery, University of Ulsan College of Medicine, Gangneung Asan Hospital, Gangneung 210-711, Republic of Korea; ^3^Department of Life and Nanopharmaceutical Science, College of Pharmacy, Kyung Hee University, Seoul 130-701, Republic of Korea; ^4^Natural Products Research Center, Korea Institute of Science and Technology, Gangneung 210-340, Republic of Korea; ^5^School of Pharmacy, Sungkyunkwan University, Suwon 440-746, Republic of Korea; ^6^Department of Pathology, University of Ulsan College of Medicine, Gangneung Asan Hospital, Gangneung 210-711, Republic of Korea

## Abstract

The present study investigated the renoprotective effect of an *Artemisia asiatica* extract and eupatilin in kidney epithelial (LLC-PK1) cells. Although cisplatin is effective against several cancers, its use is limited due to severe nephrotoxicity. Eupatilin is a flavonoid compound isolated from the *Artemisia* plant and possesses antioxidant as well as potent anticancer properties. In the LLC-PK1 cellular model, the decline in cell viability induced by oxidative stress, such as that induced by cisplatin, was significantly and dose-dependently inhibited by the *A. asiatica* extract and eupatilin. The increased protein expressions of phosphorylated JNK and p38 by cisplatin in cells were markedly reduced after *A. asiatica* extract or eupatilin cotreatment. The elevated expression of cleaved caspase-3 was significantly reduced by *A. asiatica* extract and eupatilin, and the elevated percentage of apoptotic cells after cisplatin treatment in LLC-PK1 cells was markedly decreased by cotreatment with *A. asiatica* extract or eupatilin. Taken together, these results suggest that *A. asiatica* extract and eupatilin could cure or prevent cisplatin-induced renal toxicity without any adverse effect; thus, it can be used in combination with cisplatin to prevent nephrotoxicity.

## 1. Introduction

Cisplatin is a potent chemotherapeutic agent for the treatment of multiple human malignancies [1, 2]. It accumulates in all segments of nephron but is predominantly taken up by the proximal tubule cells, which then provokes severe damage [3]. The efficacy of cisplatin is dose dependent, but the side effect in kidney limits the use of higher doses to improve its chemotherapeutic effects [4, 5]. The toxic effects of cisplatin mainly occur via oxidative stress and DNA damage [6, 7], ultimately leading to apoptotic pathways in tumour cells [8] and also in renal cells [4, 9, 10].

For centuries, many natural products have been identified for the prevention and/or treatment of kidney diseases because they are believed to have nephroprotective effects. They are widely used in clinical practice in many parts of the world. For example,* Silybum marianum* was found to attenuate nephrotoxicity induced by gentamicin in dogs [11]. A water extract of* Kalanchoe pinnata* leaves protected rat kidneys from gentamicin-induced nephrotoxicity [12]. Salviae Radix extract exerted a protective effect against cisplatin-induced renal cell injury, and its effect might be mediated by its antioxidant effect [13].

*Artemisia asiatica* Nakai is a traditional oriental medicine and it has been used for the treatment of several inflammatory disorders. Recent studies revealed that* A. asiatica* has antioxidative and anti-inflammatory effects contributing to its protective effects against various pathophysiological conditions including gastric damage [14], liver damage [15], experimental pancreatitis [16], and tumor promotion [17].

**Figure 1:**
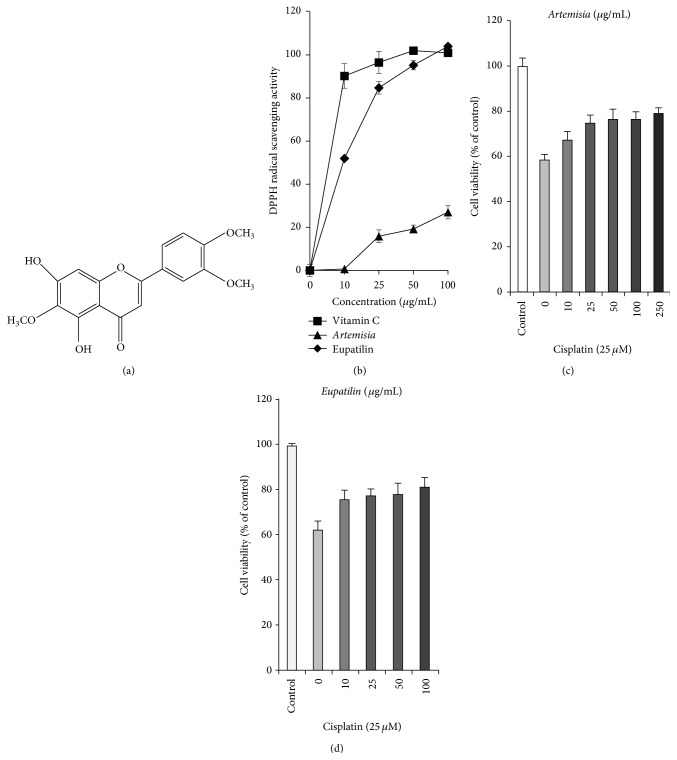
Effects of* A. asiatica* extract and eupatilin on cisplatin-induced nephrotoxicity in LLC-PK1 cells. (a) Structure of eupatilin. (b) Comparison of DPPH radical scavenging effects of* A. asiatica* extract, eupatilin, and vitamin C. (c) Dose-dependent protective effect of* A. asiatica* extract against cisplatin-induced nephrotoxicity in cells. (d) Dose-dependent protective effect of eupatilin against cisplatin-induced nephrotoxicity in cells.

Stillen is a commercially available extract from* A. asiatica*. Eupatilin (Figure 1(a)), an active compound isolated from* A. asiatica*, has been reported to treat peptic ulcers and gastritis. It has antioxidative and anti-inflammatory effects against gastric mucosal injury [18, 19]. Various inflammatory mediators such as cytokines and oxidative stress that can affect gastric mucosal injury are thought to be involved in its action mechanism [20, 21]. Eupatilin was also reported to have therapeutic potential for the treatment of gastric cancer [22, 23].

Although cisplatin-induced nephrotoxicity has been well documented, the effects of* A. asiatica* and eupatilin on apoptosis in kidney cells after cisplatin exposure remain under active investigation.

## 2. Materials and Methods

### 2.1. Chemicals and Reagents

An ethanolic extract of* A. asiatica* and its active compound eupatilin were prepared as reported previously [17, 18]. Cisplatin and 1,1-diphenyl-2-picryl-hydrazyl (DPPH) were purchased from Sigma Chemical Co. (St. Louis, MO, USA). The stock solution of chemicals was prepared in 100% dimethylsulfoxide (DMSO) and stored at −20°C until use. Antibodies for p38, p-p38, JNK, p-JNK, ERK, p-ERK, cleaved caspase-3, and GAPDH were purchased from Cell Signaling (Boston, MA, USA).

### 2.2. Protective Effect against Cisplatin-Induced Nephrotoxicity in Cells

Possible renoprotective effects against cisplatin-induced damage were evaluated in LLC-PK1 cells as reported previously [24]. In brief, LLC-PK1 cells were seeded in 96-well culture plates at 1 × 10^4^ cells per well and the test sample and/or radical donor, 25 *μ*M cisplatin, were added to the culture medium. Twenty-four hours later, the cell viability was measured by using a microplate reader (PowerWave XS; Bio-Tek Instruments, Winooski, VT, USA).

### 2.3. DPPH Radical Scavenging Assay

The radical scavenging activity of* A. asiatica* and eupatilin against DPPH was determined spectrophotometrically. In microwells, 100 *μ*L of an aqueous solution of the completely dissolved sample (control: 100 *μ*L DW) was added to an ethanolic solution of DPPH (100 *μ*L, 60 *μ*M) according to the reported method [25]. The final concentrations of the tested samples in the assayed solution were 10, 25, 50, and 100 *μ*g/mL. Vitamin C was used as the standard for comparison. The ability to scavenge DPPH radicals was calculated in terms of percentage of inhibition according to the following equation: % inhibition = [(*A*_0_ − *A*_1_)/*A*_0_ × 100], where *A*_0_ is the absorbance of the control (without extract) and *A*_1_ is the absorbance in the presence of the extract.

### 2.4. Western Blot Analysis

Proteins (whole cell extracts, 30 *μ*g/lane) were separated by electrophoresis in a precast 4–15% Mini-PROTEAN TGX gel (Bio-Rad, CA, USA) blotted onto PVDF transfer membranes as reported previously [26]. Bound antibodies were visualized using ECL Advance Western Blotting Detection Reagents (GE Healthcare, UK) and a LAS 4000 imaging system (Fujifilm, Japan).

### 2.5. Image-Based Cytometric Assay

To determine the portion of the population that had become apoptotic, cells were stained with annexin V-Alexa Fluor 488 conjugate using a Tali image-based cytometer (Invitrogen, CA, USA) [27]. Propidium iodide (PI) was used to differentiate dead cells (annexin V-positive/PI positive or annexin V-negative/PI positive) from those that were apoptotic (annexin V-positive/PI negative). The percentages of the population reported as viable, apoptotic, and dead by the Tali cytometer were comparable with data from the same samples independently run on a flow cytometer.

### 2.6. Statistical Analysis

Statistical significance was determined through analysis of variance (ANOVA). *p* values of less than 0.05 were considered statistically significant.

## 3. Results and Discussion

### 3.1. Effects of* A. asiatica* Extract and Eupatilin on Cisplatin-Induced Nephrotoxicity in LLC-PK1 Cells

The antioxidant effects of* A. asiatica* and eupatilin were tested using DPPH, a stable free radical. DPPH decolorizes in the presence of antioxidants. The scavenging ability of* A. asiatica* and eupatilin was represented by a line diagram and compared with vitamin C (Figure 1(b)). This result suggests that eupatilin is the antioxidant and active component of* A. asiatica*. As shown in Figure 1, the cell viability was decreased significantly to about 60% of that of untreated control cells (*p* = 0.0004) after 25 *μ*M cisplatin treatment. However, pretreatment with the* A. asiatica* extract and eupatilin markedly restored cell viability to 80 and 82%, respectively, in a dose-dependent manner (Figures 1(c) and 1(d)).

### 3.2. Involvement of MAPKs-Caspase-3 Signaling Pathway in the Protective Effect of* A. asiatica* Extract and Eupatilin against Cytotoxicity in Cultured LLC-PK1 Cells

**Figure 2:**
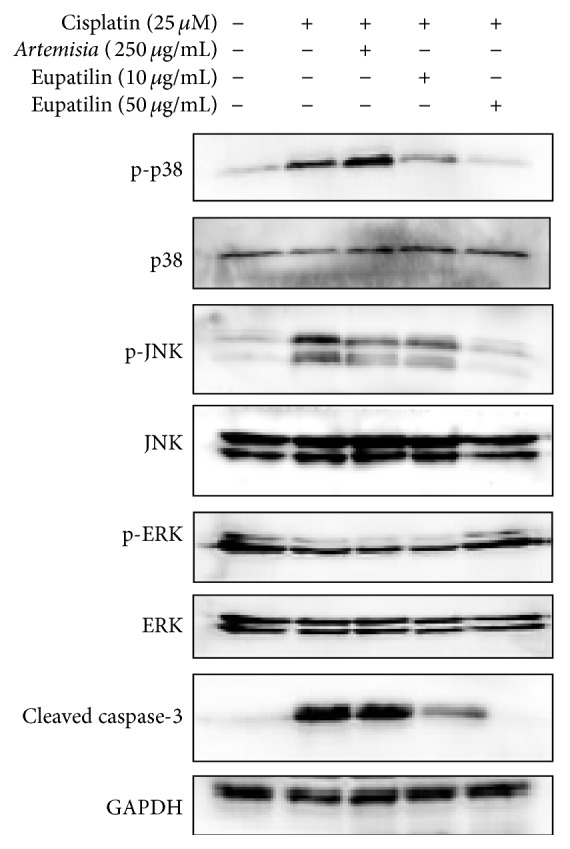
Involvement of the MAPKs-caspase-3 signaling pathway in the protective effect of* A. asiatica* extract and eupatilin against cytotoxicity in cultured LLC-PK1 cells. Results of the Western blot show the levels of p-p38, p38, p-JNK, JNK, p-ERK, ERK, and cleaved caspase-3 in LLC-PK1 cells treated with* A. asiatica* extract and eupatilin and/or cisplatin at different concentrations for 24 h. Whole cell lysates (20 *μ*g) were separated by SDS-PAGE, transferred onto PVDF transfer membranes, and probed with the indicated antibodies. Proteins were visualized using an ECL detection system.

Figure 2 shows the protein expressions of p38, p-p38, JNK, p-JNK, ERK, p-ERK, and cleaved caspase-3 after* A. asiatica* (250 *μ*g/mL) and eupatilin (10 and 50 *μ*g/mL) treatment. As shown in Figure 2, the phosphorylation of p38 and JNK was decreased in LLC-PK1 cells by* A. asiatica* and eupatilin treatments. In addition, the elevated protein expression of cleaved caspase-3 was also markedly reduced by* A. asiatica* and eupatilin treatments. ERK protein expression in the LLC-PK1 cells was slightly decreased by cisplatin treatment and increased by* A. asiatica* and eupatilin treatment; however, the differences were not of significant effect.

### 3.3. Effects of* A. asiatica* Extract and Eupatilin on Apoptosis in LLC-PK1 Cells

**Figure 3:**
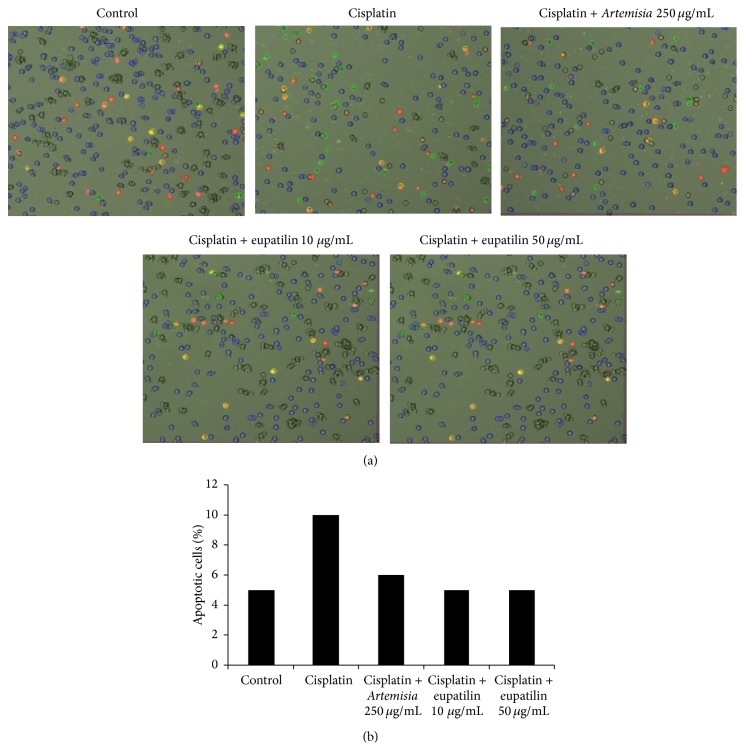
Effects of* A. asiatica* extract and eupatilin on apoptosis in LLC-PK1 cells. (a) Representative images of apoptosis detection. (b) Percentage of annexin V-positive-stained apoptotic cells. Dead and apoptotic cells were stained red and green, respectively. Apoptosis was determined by a Tali image-based cytometer.

Figure 3 shows the effects of the* A. asiatica* extract and eupatilin on apoptosis in LLC-PK1 cells. As shown in Figure 3(a), the number of dead and apoptotic cells, which were stained with red or green colors, was increased by cisplatin treatment, whereas it was decreased after cotreatment with the* A. asiatica* extract (*p* = 0.008) and more significantly eupatilin (*p* = 0.003). The elevated percentage of apoptotic cells after cisplatin treatment was markedly decreased after cotreatment with the* A. asiatica* extract and eupatilin (Figure 3(b)).

## 4. Discussion

The protective effect of the* A. asiatica* extract and eupatilin against cisplatin-induced nephrotoxicity was tested using LLC-PK1 cells, which are the most vulnerable renal tubular cells to oxidative stress [24, 28]. It is reported that reactive oxygen species (ROS) play vital biological roles in cellular homeostasis whereas the increased ROS levels are associated with apoptosis in cells [29–31]. Our result suggests that eupatilin is the antioxidant and active component of* A. asiatica*. In addition, pretreatment with the* A. asiatica* extract and eupatilin markedly ameliorated reduced LLC-PK1 cell viability by cisplatin in a dose-dependent manner.

It has been reported that ROS act as a second messenger initiating signal transduction cascades, including the MAPKs signaling pathway [32, 33]. The MAPKs are important mediators for apoptosis induction in response to anticancer drugs, in particular cisplatin [34, 35]. In the present study, the increased protein expressions of phosphorylated JNK and p38 by cisplatin in LLC-PK1 cells were markedly ameliorated after* A. asiatica* extract or eupatilin cotreatment. To further investigate the ability of* A. asiatica* and eupatilin to prevent apoptosis, we measured the expression levels of cleaved caspase-3, known as an index of apoptosis, in the kidney. As shown in the results,* A. asiatica* and eupatilin significantly reduced the expression of cleaved caspase-3. These results suggest that cisplatin-induced increases in the ROS level activate MAPKs in LLC-PK1 cells, whereas the* A. asiatica* extract and eupatilin inhibit the activation of p38 and JNK.

Cisplatin-induced renal cell damage is dependent on apoptosis induced by DNA damage [36, 37]. Apoptosis in renal tubular cells has been observed in several kinds of renal disorders [38]. The elevated protein level of cleaved caspase-3 decreased after treatment with* A. asiatica* extract and eupatilin.

In conclusion, our results suggest that the* A. asiatica* extract can ameliorate nephrotoxicity in LLC-PK1 cells. Eupatilin serves as one of the major components by blocking the MAPKs-caspase-3 signaling cascade.
